# Template-Assisted Formation of Nanostructured Dopamine-Modified Polymers

**DOI:** 10.3390/nano7110364

**Published:** 2017-11-02

**Authors:** Liping Zhu, Takashi Isoshima, Baiju G. Nair, Yoshihiro Ito

**Affiliations:** 1 Nano Medical Engineering Laboratory, RIKEN, 2-1 Hirosawa, Wako, Saitama 351-0198, Japan; lpzhu@riken.jp (L.Z.); isoshima@riken.jp (T.I.); bgnair@riken.jp (B.G.N.); 2 Emergent Bioengineering Materials Research Team, RIKEN Center for Emergent Matter Science, 2-1 Hirosawa, Wako, Saitama 351-0198, Japan

**Keywords:** dopamine, template, nanostructure, modified polymer, catechol

## Abstract

Dopamine-modified alginate and gelatin were prepared. The polymers were characterized and the properties of their aqueous solutions were investigated. Aqueous solutions of dopamine-modified alginate and gelatin with a concentration exceeding 20 mg/mL naturally formed gels after 16 h. Although polydopamine itself was not used for template-assisted nanostructure formation, the modified polymers could be used with dopamine. Mixing with dopamine allowed the precise shape of the template to be maintained in the resulting material, allowing nanopatterned surfaces and nanotubes to be prepared.

## 1. Introduction

In 2007, it was reported that dopamine can be used to modify various surfaces including both inorganic and organic ones [1] using a surface coating method that involves dipping the substrate in a slightly alkaline dopamine solution. Because this method is simple, it has been used in many applications including biocompatible materials, sensing materials, energy conversion, catalysts, and environmental cleanup [2,3,4,5,6,7,8,9,10,11,12,13,14,15,16,17,18,19,20,21]. In this method, dopamine not only coats the surface but also forms a polydopamine (PDA) film on the surface followed by oxidative reaction. Therefore, PDA has also been used to produce hollow capsules at the solid/liquid interface [22] using sacrificial silica templates or from the liquid/liquid interface of an oil emulsion in water [23,24,25] for drug delivery [25]. However, PDA tends to form grains and is brittle. 

Some modifications have been devised to circumvent the mechanical fragility of PDA films. The first approach is layer-by-layer formation [26,27], and the second is “composite” PDA-based films. Polymers carrying amino groups in the subphase containing dopamine are able to chemically cross-link PDA during its formation, leading to composite PDA-based films [28,29,30,31,32]. The third method is synthesis of dopamine-derivatized polymers. The fourth is appending catechol moieties onto polymers such as poly(acrylic acid) [27], polyethylene glycol [33], chitosan [34], alginate [35], and gelatin [36] to generate 3D hydrogel networks followed by the oxidative cross-linking of the catechol groups. 

In this study, dopamine-modified alginate and gelatin were prepared. The polymers were characterized by oxidation, gelation, and film formation. Finally, template-assisted nanostructures were prepared using these modified polymers. 

## 2. Materials and Methods

### 2.1. Materials

Chemicals were used as received without further purification. Dopamine hydrochloride and gelatin (from porcine skin Type A) were purchased from Sigma-Aldrich (St. Louis, MO, USA). Sodium alginate (80–120 mPa·s) was purchased from Wako Pure Chemical Industries, Ltd. (Osaka, Japan). *N*-hydroxysuccinimide (NHS) and 1-(3-dimethylaminopropyl)-3-ethylcarbodiimide hydrochloride (EDC) were purchased from Tokyo Chemical Industry Co., Ltd. (Tokyo, Japan).

### 2.2. Polymer Modification

**Scheme 1:**
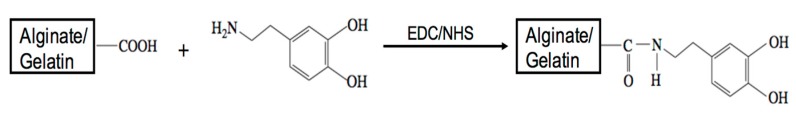
Schematic illustration of dopamine conjugation to alginate and gelatin.

The preparation process of the dopamine-modified polymers is shown in Scheme 1. The alginate-catechol (AC) conjugate was synthesized by chemical reaction using EDC and NHS. The modification was performed according to previous method [35]. Sodium alginate was dissolved in Dulbecco’s phosphate buffered saline (DPBS, pH = 4.5) at a concentration of 1% (*w*/*v*). EDC and NHS were then added to the alginate solution at a molar ratio equal to that of alginate and reacted at room temperature for 15 min. Dopamine hydrochloride was added to the solution at a 3:1 molar ratio of catechol groups to carboxyl groups. The reaction was performed at room temperature for 12 h. The reaction mixture was dialyzed using DPBS (pH = 4) and acidic distilled water (pH = 5) for 24 h with a dialysis membrane (Spectra/Por, molecular weight cutoff 12,000–14,000), and subsequently freeze-dried. Gelatin was dissolved in DPBS (pH = 4) at 40 °C, and then a gelatin-catechol (GC) conjugate was synthesized using the method described above. 

Syntheses of AC and GC were confirmed by ^1^H nuclear magnetic resonance (NMR) measurements on a 400 MHz NMR spectrometer (JEOL AL400, JEOL Ltd., Tokyo, Japan). The substitution degrees of catechol groups for carboxyl groups in AC and GC were determined by measuring the absorbance at 280 nm using a Ultraviolet-visible (UV–vis) spectrophotometer (V-550, JASCO Co., Tokyo, Japan) (scan speed, 200 nm/min; response, medium; bandwidth, 1.0 nm) because the aromatic ring structure of conjugated catechol group exhibits an absorption peak at this wavelength [35]. Dopamine hydrochloride in DPBS (pH = 4.5) was used to generate a standard curve for catechol concentrations ranging from 0.125 to 0.00195 mg/mL by 1:2 serial dilution. AC (0.25 mg/mL) and GC (1 mg/mL) were used as samples; the contribution of gelatin (1 mg/mL) was subtracted. The substitution degrees of catechol groups for carboxyl groups in AC and GC were also estimated using NMR spectroscopy. The absolute weight of catechol in polymers was calculated based on the absorbance measurements.

### 2.3. Time Course of Oxidation of Conjugates and Composites

AC and GC (2, 10, 20, 40, and 60 mg) were dissolved in 10 mM Tris-HCl buffer (2 mL, pH = 8.5) to make solutions with various concentrations. The solutions were incubated at room temperature, and photographs were taken after prescribed periods.

### 2.4. Films Formed on Membranes and Thickness Measurement

AC and GC (5 mg/mL), dopamine hydrochloride (1 mg/mL), and a total of 1 mg/mL of GC mixed with dopamine hydrochloride at weight ratios of 10:1 (denoted as GC-dopamine 10:1) and 1:1 (denoted as GC-dopamine 1:1) were dissolved in 10 mM Tris-HCl buffer (pH = 8.5). Polycarbonate membranes (HTTP02500, Merck Millipore Ltd., Carrigtwohill, County Cork, Ireland) were dipped in the solutions. After incubating at 60 °C for 8 h (7 h for GC-dopamine 10:1 and GC-dopamine 1:1) at a shaking speed 100 rpm, the membranes were rinsed with the same buffer several times to remove the aggregated particles and then washed at 40 °C three times at a shaking speed 80 rpm for 10 min each time. The membranes were then dried under low vacuum conditions.

Glass substrates (diameter: 15 mm) were cleaned by sequential sonication in 6 M HCl and then acetone for 10 min each time. Ultrapurified water (20 µL) from Milli-Q purifier (Millipore SAS, Molsheim, France) was then dropped on each glass substrate and a piece of polycarbonate membrane (around 2 mm × 3 mm rectangle) coated with AC, GC, dopamine, GC-dopamine 10:1, or GC-dopamine 1:1 was placed on the Milli-Q drop with the film facing the glass. The glass was then dried in air. Dimethylformamide (DMF) (100 µL) was dropped on the membrane and incubated at room temperature for around 5 min to dissolve the polycarbonate membrane before discarding; this dissolving process was repeated six times. Finally, the DMF was removed by rinsing the film with acetone and drying in air. The thickness of the film transferred to the glass substrate was measured with a reflection confocal laser microscope (OLS4100, Olympus, Tokyo, Japan) by scratching the film with tweezers to expose the glass substrate.

### 2.5. Template-Assisted Nanopattern Film Fabrication

**Scheme 2:**
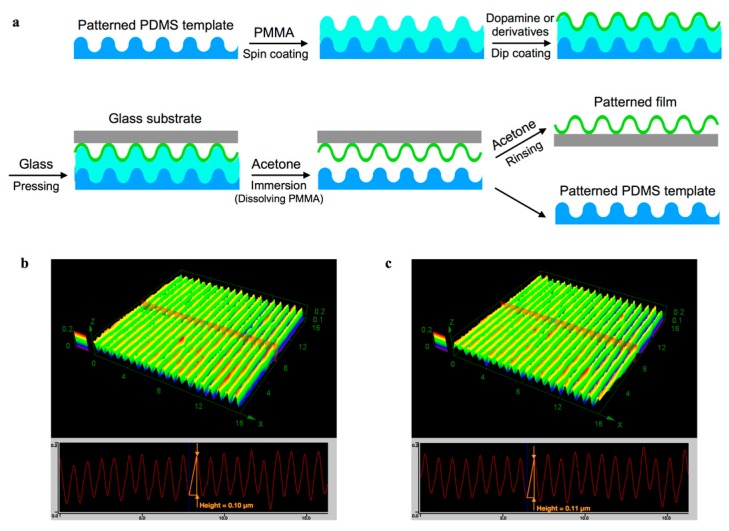
(**a**) Illustration of template-assisted patterned film fabrication. Morphology of a Polydimethylsiloxane (PDMS) template; (**b**) before and (**c**) after spin coating with poly(methyl methacrylate) (PMMA), captured by a reflection confocal laser microscope.

The nanopattern film fabrication process is illustrated in Scheme 2. The poly(methyl methacrylate) (PMMA) template was prepared by the following process. Polydimethylsiloxane (PDMS) templates with a surface grating transferred from an optical ruled grating of 1200 groove/mm pitch were cut into small pieces of 1.5 cm × 1.5 cm. A sacrificial layer of PMMA was formed on PDMS by spin-coating PMMA solution (200 µL, 10 mg/mL) at rotational speed of 500 rpm for 5 s followed by 5000 rpm for 45 s. The thickness of the PMMA layer was about 300 nm. Four solutions of dopamine hydrochloride, GC, and their mixtures were prepared in 10 mM Tris-HCl buffer (pH = 8.5) to give a final concentration of 1 mg/mL. These solutions were prepared with two different ratios of dopamine to dopamine-modified polymers. The PDMS/PMMA templates were dipped in the solutions and incubated at 60 °C for 12 h at a shaking speed of 100 rpm. The templates were rinsed with the same buffer several times to remove the aggregated particles and then washed at 40 °C three times at a shaking speed of 80 rpm for 10 min each time. After brief drying in air, glass substrates hydrophilized by ozone treatment for 10 min were placed on the templates and gently pressed to increase their contact. The samples were immersed in acetone for 30 min. The glass substrates were picked up gently and rinsed with acetone for 1 min. The surface morphology and thickness of the patterned films were examined by the reflection confocal laser microscope. 

### 2.6. Template-Assisted Nanotube Fabrication

**Scheme 3:**
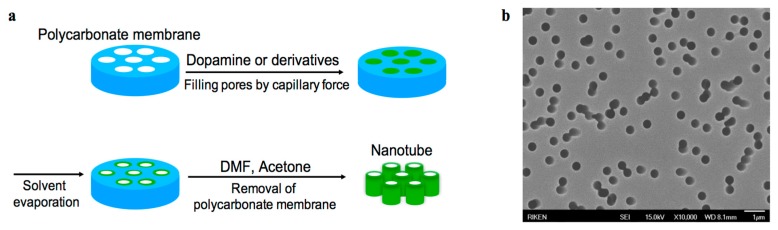
(**a**) Illustration of template-assisted nanotube fabrication using a porous polycarbonate membrane; (**b**) scanning electron microscopy (SEM) image of the polycarbonate membrane used as a template.

As shown in Scheme 3, a three-step process was used to prepare the nanotubes. Glass substrates (diameter: 15 mm) were cleaned by sequential sonication in 6 M HCl and then with acetone for 10 min each time. Four solutions of dopamine, dopamine-modified polymer, and their mixtures were prepared in 10 mM Tris-HCl buffer (pH = 8.5) to give a final concentration of 1 mg/mL. Solutions with two different ratios of dopamine to dopamine-modified polymers were prepared. A polycarbonate membrane as described above (pore diameter: 400 nm, thickness: 10 μm) was placed on top of each solution drop, causing the solution to rise into the nanopores of the membrane by capillary force [37]. After solvent evaporation under ambient conditions, thin films were deposited onto the walls of nanopores in the membrane, resulting in the formation of AC-dopamine nanotubes. These nanotubes were further dried at 60 °C under vacuum for 18 h to remove the residual solvent. The polycarbonate template was then dissolved using DMF and washed with acetone as described above. The nanotubes remaining on the glass substrate were examined by scanning electron microscopy (SEM; JSM6330F, JEOL Ltd., Tokyo, Japan), after forming a Pt–Pd coating of circa 30 nm to increase conductivity.

## 3. Results and Discussion

### 3.1. Polymer Synthesis

**Figure 1:**
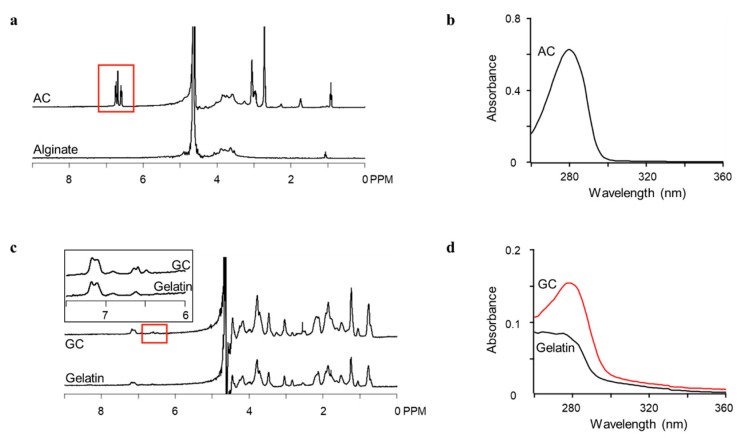
^1^H nuclear magnetic resonance (NMR) analysis of (**a**) Alginate-catechol (AC) and (**c**) Gelatin-catechol (GC), the inset in (**c**) shows the enlarged spectra from 6 to 7.5 ppm. Ultraviolet-visible (UV–vis) spectra of 0.25 mg/mL AC (**b**) and 1 mg/mL GC and gelatin (**d**).

AC and GC were synthesized by standard carbodiimide coupling chemistry using EDC and NHS. In this reaction, the carboxyl groups of alginate and gelatin were activated by EDC/NHS and the amine group of dopamine was then coupled with the activated carboxyl group (Scheme 1). The reaction was carried out in an aqueous buffer (pH = 4.5) with a molar ratio of dopamine to carboxyl groups of alginate or gelatin of 3:1. The alginate-catechol (AC) conjugation and gelatin-catechol (GC) conjugation were confirmed by ^1^H NMR analysis as shown in Figure 1a,c respectively; the peaks of catechol protons appeared at around 6.8 ppm (highlighted with red boxes). The substitution degrees of catechol groups for carboxyl groups in AC and GC determined by their absorbance at 280 nm using a UV–vis spectrophotometer were almost the same, consistent with the results determined by ^1^H NMR analysis (Figure 1 and Table 1). The substitution degree of each product depended on the kind of polymer. Gelatin has many kinds of functional groups other than carboxyl groups, which might hinder the coupling reactions. The catechol weights in the polymers were calculated based on absorbance measurements (Table 1). 

In the case of alginate derivatives, previous reports obtained catechol contents ranging from 5% to 15% using a feed ratio of carboxylate to catechol of 1:1 [32,35,38]. Because this study used a 3:1 ratio, a higher content of catechol in the alginate was achieved. In contrast, the catechol content of the gelatin derivative was almost the same as that in a previous report [36].

### 3.2. Oxidation and Gelation of Conjugates and Composites

**Figure 2:**
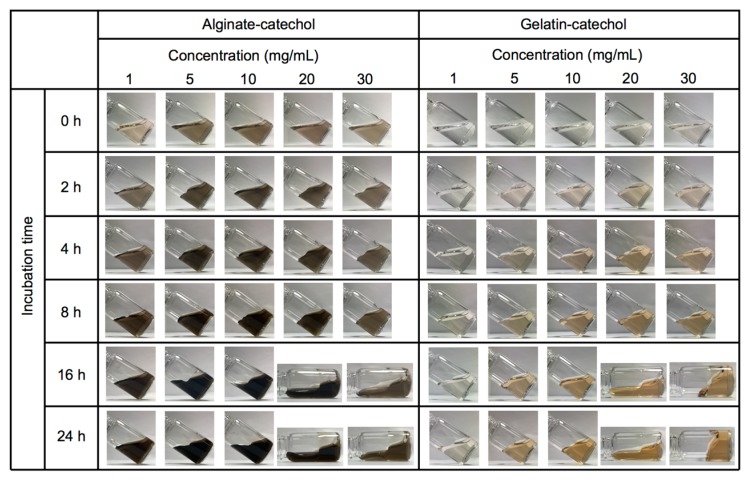
Photographs of gelation of AC and GC at various concentrations.

The changes of aqueous solutions containing the prepared conjugates are illustrated in Figure 2. Various concentrations of AC and GC were dissolved in 10 mM Tris-HCl buffer (pH = 8.5); gel formation was observed at concentrations over 20 mg/mL after 16 h for both AC and GC. In a previous report, above an AC concentration of 30 mg/mL, a hydrogel formed in the presence of dopamine [32]. Additionally, it has also been reported that cross-linking of AC was accelerated to form a hydrogel under basic conditions in the presence of the chemical oxidizing agent sodium periodate (NaIO_4_); equimolar amounts of NaIO_4_ and catechol in AC resulted in the formation of a hydrogel within 3–4 min after induction of gelation [35]. The hydrogel formation of GC has not been reported before.

### 3.3. Film Formation

**Figure 3:**
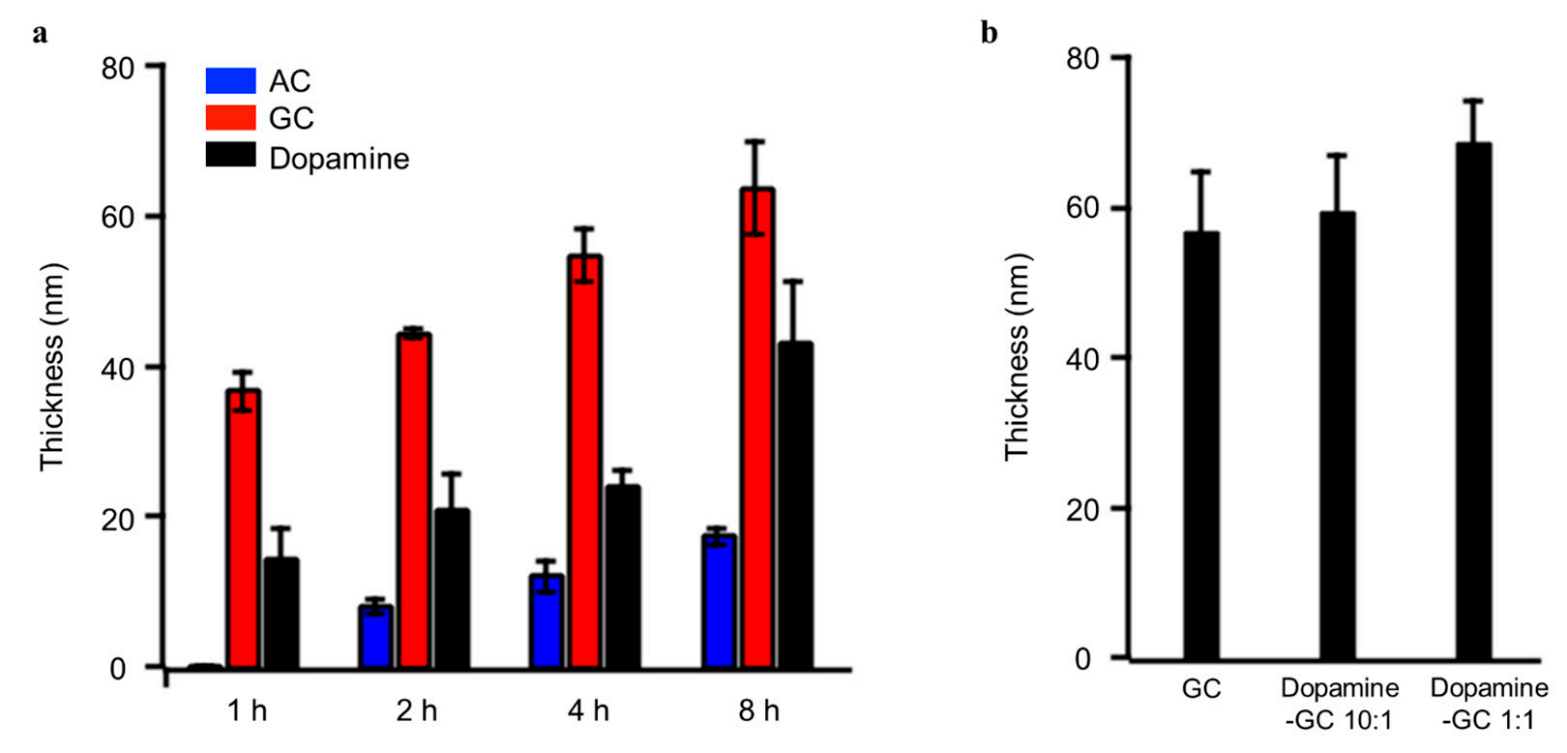
(**a**) Thicknesses of films formed from AC and GC (5 mg/mL) and dopamine hydrochloride (1 mg/mL) at various incubation times on polycarbonate membranes and then transferred to glass surfaces; (**b**) Thicknesses of films formed from 1 mg/mL of GC, GC-dopamine 10:1, and GC-dopamine 1:1 in 7 h on polycarbonate membranes and then transferred to glass surfaces.

Films were formed using concentrations that did not cause gel formation, and their thicknesses were measured. Substrates were dipped in AC and GC (5 mg/mL), and dopamine (1 mg/mL) was used as a control (Figure 3a). After incubating for various periods, the substrates were washed and the films formed on the membranes were transferred to glass surfaces. Film thickness increased over time. GC formed thicker films than AC even though its content of catechol was lower than that of AC.

Substrates were also dipped in GC (1 mg/mL), and a total concentration of 1 mg/mL of GC mixed with dopamine at weight ratios of 10:1 (denoted as GC-dopamine 10:1) and 1:1 (denoted as GC-dopamine 1:1) (Figure 3b). After incubating for 7 h, the substrates were washed and the films formed on the membrane were transferred to glass surfaces. GC films of similar thickness formed in the presence and absence of dopamine. Thus, dopamine did not strongly affect film formation.

### 3.4. Template-Assisted Nanopattern Film Formation

**Figure 4:**
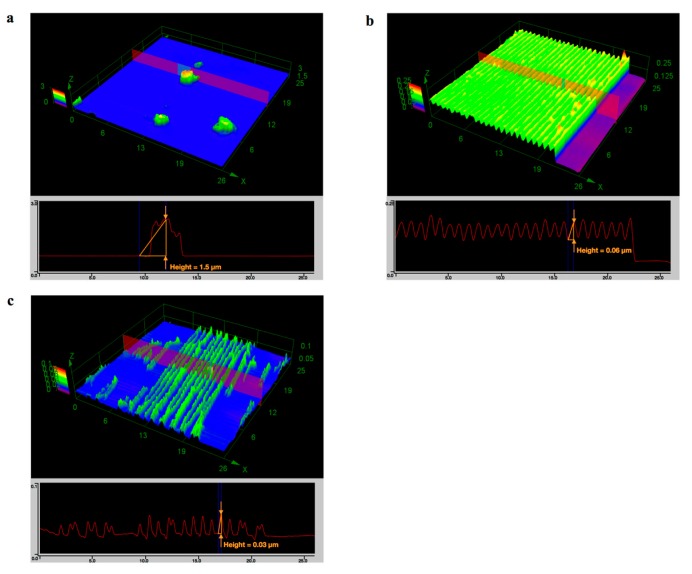
Morphologies of patterned films fabricated using PDMS templates with surface gratings visualized by a reflection confocal laser microscope. (**a**) Dopamine hydrochloride alone; (**b**) GC–dopamine 1:1; and (**c**) GC alone. The measured area was 26 µm × 26 µm. The height scale was (**a**) 3 µm and (**b**) 0.25 µm and (**c**) 0.1 µm, and the vertical axis in the 3D images in (**b**,**c**) were magnified 50×. Film edges were intentionally selected for observation to show the exposed substrate surface.

The morphologies of the patterned films produced under each sets of conditions are shown in Figure 4. For dopamine alone, only large grains were found on the glass substrate; no film was observed. This is because the surface of the film formed on the PDMS/PMMA template was rough with large particles, so the film did not easily contact the glass substrate and thus was not transferred cleanly (Figure 4a). In contrast, the film formed from a mixture of dopamine and GC (GC-dopamine 1:1) provided a grating pattern with the same period as that of the template (Figure 4b). In Figure 4b, the edge of the film was shown so that the exposed substrate was also observable. In the film area, the top part was about 140 nm thick and the valley part was about 80 nm thick. Assuming that the film thickness is uniform, the top part has a channel structure with a difference of about 60 nm between the film and substrate. In the case of pristine GC (Figure 4c), the valley part of the film was at the same height as the glass substrate, meaning that the substrate surface was exposed. The GC-dopamine 10:1 mixture also provided a similar structure (data not shown). This suggests that although the channel structure formed, the top part of the channel was removed during the transfer and rinsing process. A higher concentration of dopamine can enhance polymerization and crosslinking, resulting in a more rigid structure. This is consistent with our finding that only GC-dopamine 1:1 preserved the channel structure.

### 3.5. Template-Assisted Nanotube Array Formation

**Figure 5:**
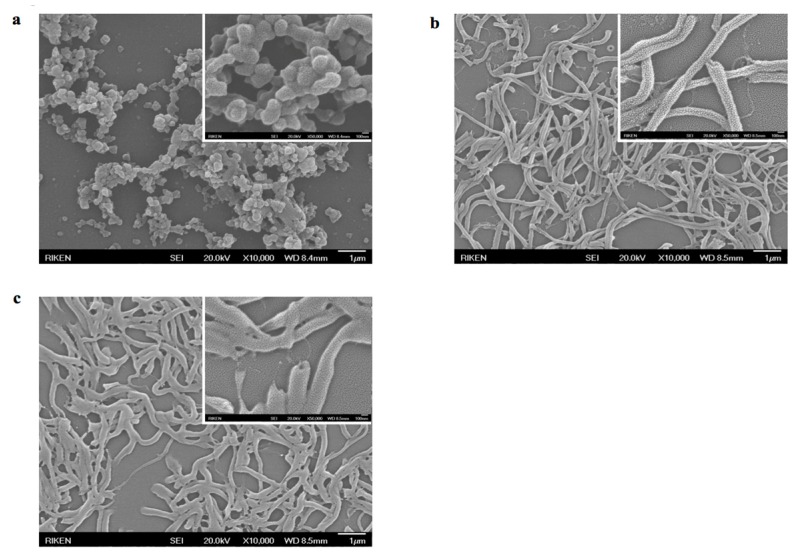
SEM images of nanotubes formed using (**a**) dopamine hydrochloride alone; (**b**) AC-dopamine 1:1; and (**c**) AC alone with the assistance of polycarbonate membranes. The insets in (**a**–**c**) are images with higher magnifications.

Figure 5 depicts nanotubes obtained from AC and dopamine mixtures. In the case of dopamine alone, no nanotubes were observed (Figure 5a). It was considered that PDA precipitated and aggregated under the membrane rather than attaching to the wall of the membrane pores. AC alone and the AC-dopamine 1:1 mixture formed nanotubes. Conversely, the GC-dopamine mixture did not form nanotubes under any of the conditions we tried. This is possibly because the high viscosity of the solution made it difficult to fill the membrane pores. Previously, we formed nanotubes through layering of anionic and cationic polypeptides with the application goal of drug delivery to cells [39]. The PDA-based nanotubes obtained here should also contribute to such applications in the future.

## 4. Conclusions

Two dopamine-modified polymers were synthesized by EDC/NHS chemistry and their oxidation, gelation, and film formation characteristics were determined. With the assistance of templates, patterned films with a channel structure and nanotube arrays were fabricated by combining dopamine with the dopamine-modified polymers. These combinations provided sufficient stiffness and flexibility to form various templated nanostructures, which was difficult with either dopamine or a dopamine-modified polymer alone. The present material design will enable us to fabricate various nanostructures for specific applications.

## Figures and Tables

**Table 1:** Composition of dopamine-modified polymers.

Dopamine-Modified Polymers	Molar Ratio in Feed Carboxyl:Catechol	Substitution Degree of Catechol Group in Carboxyl Group of Polymer		Weight of Catechol in Polymer ^3^
		UV–vis ^1^	NMR ^2^	
Alginate-catechol (AC)	1:3	22%	29%	18.5%
Gelatin-catechol (GC)	1:3	3.7%	2%	0.6%

^1^ Determined from absorbance at 280 nm measured by UV–vis spectroscopy. ^2^ Determined by NMR spectroscopy. ^3^ Calculated from ^1^.
